# Responses of soil health to seasonal change under different land cover types in a sub-tropical preserve ecosystem

**DOI:** 10.1371/journal.pone.0318092

**Published:** 2025-03-25

**Authors:** Noel Manirakiza, Suraj Melkani, Abul Rabbany, Natalia Medina-Irizarry, Samuel Smidt, Anna Braswell, Willm Martens-Habbena, Jehangir H. Bhadha

**Affiliations:** 1 Department of Soil, Water, and Ecosystem Sciences, University of Florida, Everglades Research and Education Center, Belle Glade, Florida, United States of America,; 2 School of Forest, Fisheries, and Geomatics Sciences, University of Florida, Gainesville, Florida, United States of America,; 3 American Farmland Trust, Washington, District of Columbia, United States of America,; 4 Florida Sea Grant, University of Florida/IFAS, Gainesville, Florida, United States of America,; 5 Department of Microbiology and Cell Science, Fort Lauderdale Research and Education Center, Institute of Food and Agricultural Sciences, University of Florida, Davie, Florida, United States of America; Qingdao Agricultural University, CHINA

## Abstract

In subtropical preserve ecosystems, natural factors combined with anthropogenic activities have led to significant seasonal changes, including distinct dry and rainy seasons. These changes can potentially impact soil health indicators, which are keystone properties that control ecosystem services across terrestrial landscapes. Few studies have evaluated the impact of seasonal changes on soil health within non-agronomic landscapes, such as preserves. As part of this study, we collected topsoil samples (0-15 cm) from twenty-three land cover types within a 109 km² preserve in central Florida during two different seasons (dry and wet) to advance the understanding of how soil health responds to seasonal changes and to explore the environmental factors controlling soil health within non-agronomic landscapes. Ten soil indicators were analyzed and incorporated into the total dataset (TDS). From the TDS, a minimum dataset was derived using Principal Component Analysis, which was then used to calculate the Soil Health Index (SHI) for soil health assessment. Our findings showed that changes in soil indicators, their relationships, and the SHI across seasons depend on land cover type. Based on soil health classification grades, soil health status either improved, declined, or remained constant between seasons, depending on land cover type. The regression analysis of eight selected environmental factors, such as soil profile moisture (SPM), surface soil wetness (SSW), precipitation (P), soil temperature (T), elevation (El), slope gradient (S), global horizontal irradiance (GHI) and surface albedo (ALB), showed that only slope gradient significantly explains variations in SHI during wet season, whereas other environmental factors do not show significant explanatory power for SHI variations in either dry or wet season. These findings highlight the dominant influence of slope gradient on soil health within non-agronomic landscapes, while indicating that other evaluated environmental factors may have limited relevance in this context. Furthermore, the non-significant findings among soil indicators across seasons may be attributed to the study’s small sample size (i.e., three replications), a limitation stemming from constrained funding. This highlights the importance of future research incorporating larger sample size to validate the findings of this study.

1. Introduction

Soil health is defined as the ability of a living soil to function within natural or managed ecosystem boundaries to maintain plant and animal productivity, while preventing soil degradation [1]. Healthy soils are considered a steppingstone for productive ecosystems [2] and possess all physical and bio-chemical soil attributes essential for promoting and sustaining agricultural production or ecosystem structure with trivial environmental deterioration [3]. When soil health is high, the responses of plant production are maximized with a minimal soil deterioration [3]. Conversely, poor soils may be susceptible to environmental deterioration via (i) wind and water erosion, and (ii) leaching of nutrients into groundwater [3]. Recent studies reported that poor soil health is associated with depleted soil organic matter (OM) pool and degraded soil fertility [4], decreased economic growth, particularly in the countries with economy driven by agriculture [5], endangered sustainable agricultural production [6], and exacerbated human health issues associated with polluted rivers and streams [7]. Over a period from 1950 to 2010, poor soil health reduced ecosystem services by 60% in developing countries [8]. Thus, maintaining and improving soil health is vital in sustaining multiple ecosystem services (i.e., provisioning, regulating, and supporting services).

Usually, soil health is considered an indicator of soil functions [9], and its assessment is a useful tool to determine whether a given terrestrial ecosystem is either enhancing, remaining constant, decreasing, or degrading [10,11]. Performing soil health assessment (SHA) in different terrestrial ecosystems is essential for determining soil functioning, such as nutrient cycling, vegetation growth, and the sustainability of ecosystems [12]. SHA is achieved by analyzing soil properties followed by integrating these soil properties into one single index known as soil health index (SHI) [12]. This SHI is theoretically used for assessing sustainable land conservation practices, and ecosystem sustainability [13]. SHI also is used as diagnostic strategy to evaluate soil functions [13]. Since soil health improvement is a keystone to sustaining multiple terrestrial ecosystem services, understanding how seasonal change affect soil health in crop, forest, wetland, rangeland, and urban terrestrial ecosystems is vital to decision-makers for ensuring the sustainability of terrestrial ecosystem services at global scale.

Seasonal change such as infrequent rainfall periods, the often warm, and cyclic dry and rainy season are prevailing in the Tropics, Mediterranean, and Semi-arid ecosystems [14,15]. In the upcoming decades, more terrestrial ecosystems like tropical ecosystem [16–19], and savanna ecosystem [20,21] will likely be prone to seasonal change, such as prolonged and non-predictable dry and wet seasons [22]. Seasonal change has significant impact on soil health through influencing soil physical, chemical and biological properties [23]. For instance, changes in rainfall patterns significantly impact surface moisture availability [23], leading to increased nutrient leaching [24], and alterations in microbial activities associated with OM, and nutrient mineralization, thereby affecting overall soil health. Previous studies have extensively evaluated the effects of these patterns of dry and wet season on soil carbon (C) and nitrogen (N) mineralization [14,25–28], microbial biomass and respiration [29–30], soil microbiome [14,31–34], phosphorous dynamics [35], soil OM decomposition [36], and soil physical properties [30–37] within agronomic ecosystems. While extensive research has been conducted on soil health in agronomic ecosystems, studies focusing on non-agronomic landscapes, particularly in the context of seasonal change, are scarce. This lack of knowledge presents a critical research gap, particularly in understanding how diverse plant communities and land cover types within natural preserves contribute to soil health across different climatic conditions (i.e., dry, and rainy seasons). Additionally, the interaction between soil health indicators and environmental factors such as rainfall, soil moisture, temperature, elevation, slope gradient, solar radiation, and surface albedo remain underexplored in these settings.

To address this research gap, the present research was carried out at a designated natural preserve site in central Florida, characterized by seasonal change (dry season–low rainfall event, and wet season–high rainfall events) to achieve the following objectives: (1) to determine (i) soil indicators and correlation among these indicators, and (ii) how soil health changes between dry and wet season under diverse plant communities/land cover types within a natural preserve, and (2) to explore the sensitivity of soil health to environmental factors (rainfall, soil moisture, temperature, elevation, slope gradient, and solar radiation) with a view of understanding factors controlling soil health within non-agronomic landscapes. The awareness of how seasonal change shape soil health will help decision-makers to develop sustainable conservation practices for ensuring the sustainability of terrestrial ecosystem services in Sub-tropical Preserve Ecosystems.

## 2. Materials and Methods

### 2.1. Site description

**Fig 1:**
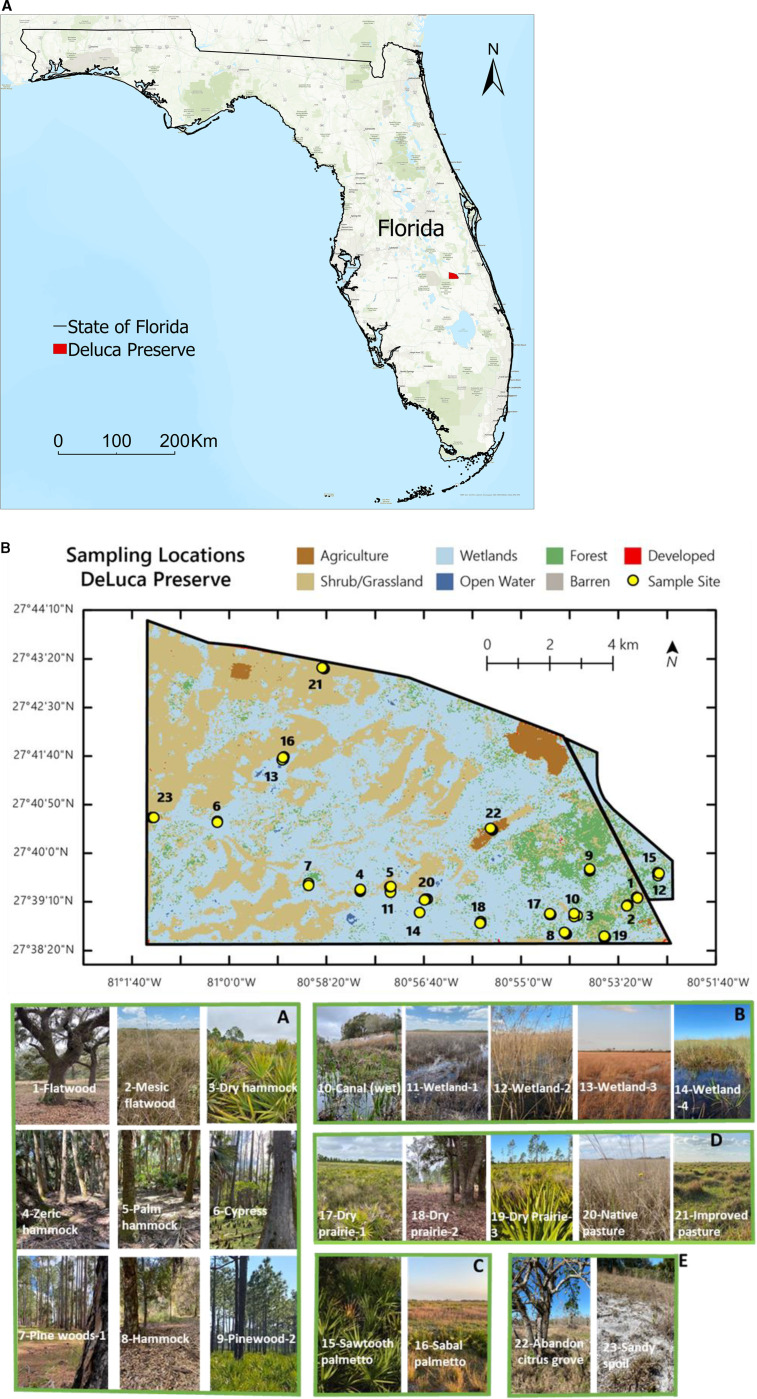
(A) Geographical location of the Deluca Preserve as the study area. (B) 1B. Geographical layout of the study area and 23 sampled land cover types with their images. Land covers were grouped into 5 ecosystem groups as follows: A: Upland forest ecosystem, B: Aquatic and wetland ecosystem, C: Shrub ecosystem, D: Range areas ecosystem, and E: Managed and barren ecosystem.

This research was conducted within the 109 km^2^ Deluca Preserve Ecosystems, located in central Florida (27^o^38′ 20′′ to 27 ^o^ 44′ 10′′ N, and 80 ^o^ 51′ 40′′ to 81 ^o^ 1′ 40′′ W) (Fig. 1A). The climate is sub-tropical with respective average annual high and low temperatures of 28.89°C, and 17.22°C, and mean yearly rainfall of approximately 1, 204 mm [38]. Deluca Preserve Ecosystems is characterized by seasonal change, including the wet rainy season (i.e., periods of high rainfall amount) from June, July, August, and September and dry cool winter season (i.e., periods of low rainfall amount) from October, November, December, January, February, March, April, and May (A1 in S1 file).

The treatments were 23 land cover types situated in Deluca Preserve Ecosystems, which were grouped into five different ecosystems (Fig. 1B). Table 1 summarizes soil and vegetation cover characteristics for each land cover type. We collected soil samples from 23 land cover types (Fig. 1B, Table 1 ) for comprehensively understanding how soil indicators, and overall soil health respond to seasonal change.

**Table 1:** Categorization of the 23 land cover types present at the Deluca Preserve Ecosystems.

S. No.	Land cover/ Ecosystem	Vegetation Cover	Soil Order	Drainage Class	Texture	Moisture Subclass	Ecosystem groups
1	Flatwood	Oak (*Quercus virginiana*)	Spodosols	PD	Sandy	Aeric	**Group A** **Upland Forests** (Forested Ecosystems/ Natural Tree Cover)
2	Mesic flatwood	Saw palmetto (*Serenoa repens*)/Gall berry (*Ilex glabra*)/ Slash pine *(Pinus elliottii)*	Spodosols	PD	Sandy	Aeric	
3	Dry hammock	Sabal palm (*Sabal palmetto*)/Live oak *(Quercus virginiana)*	Alfisols	VPD	Loamy	Typic	
4	Xeric hammock	Live oak *(Quercus virginiana)*/ Sabal palm (*Sabal palmetto*)	Alfisols	PD	Loamy	Typic	
5	Palm hammock (Xeric)	Oak (Quercus *sp.*)	Spodosols	PD	Sandy	Aeric	
6	Cypress	Natural tree cover	Spodosols	PD	Sandy	Aeric	
7	Pinewood-1	Slash pine *(Pinus elliottii)*/Sabal palm (*Sabal palmetto*)	Entisols	PD	Sandy	Typic	
8	Hammock	Oak (*Quercus virginiana*), pines (*Pinus*), palmettos	Spodosols	PD	Sandy	Aeric	
9	Pinewood-2	Pine (*Pinus*)/saw palmetto (*Serenoa repens*)/Gall berry (*Ilex glabra*)	Spodosols	PD	Sandy	Aeric	
10	Canal (wet)	Cattails (*Typha*)/Pickerelweed (*Pontederia cordata*)	Alfisols	VPD	Loamy	Typic	**Group B** **Wetland Ecosystems** (Wetland, Watercourse, and Waterbody Areas)
11	Wetland-1	Aquatic plants	Entisols	VPD	Sandy	Typic	
12	Wetland-2	Aquatic plants, 15-inch depth	Entisols	VPD	Loamy	Typic	
13	Wetland-3	Juncus*/*hypericum/Panicum	Alfisols	VPD	Loamy	Typic	
14	Wetland-4	Sawgrass (*Cladium*), 8-inch depth	Entisols	VPD	Loamy	Typic	
15	Sawtooth palmetto	Sawtooth palmetto (*Serenoa repens*)/Gall berry (*Ilex glabra*)	Entisols	VPD	Loamy	Typic	**Group C** **Shrub Ecosystems**
16	Sabal palmetto	Saw palmetto (*Serenoa repens*)/Sabal palm (*Sabal palmetto*)/black berry (*Rubus sp.*)	Alfisols	VPD	Loamy	Typic	
17	Dry Prairie-1	Gall berry (*Ilex glabra*)/Saw palmetto (*Serenoa repens*)/Running oak (*Quercus sp.*)	Entisols	PD	Sandy	Typic	**Group D** **Range Areas** (Grassland and Prairie Ecosystems)
18	Dry Prairie-2	Andropogon (*Andropogon*)/wiregrass (*Aristida stricta*)	Entisols	PD	Sandy	Typic	
19	Dry Prairie-3	Slash pine *(Pinus elliottii)*/Saw palmetto (*Serenoa repens*)	Spodosols	PD	Sandy	Aeric	
20	Native pasture	Panic grass (*Panicum)*	Spodosols	PD	Sandy	Aeric	
21	Improved pasture	Bahia grass (*Paspalum notatum*)/Andropogon (*Andropogon*)	Spodosols	PD	Sandy	Aeric	
22	Abandoned citrus grove	Abandoned Citrus Plantation	Spodosols	PD	Sandy	Aeric	**Group E** **Barren Ecosystems**
23	Sandy spoil	No vegetation	Entisols	PD	Loamy	Typic	

PD: Poorly drained; VPD: Very poorly drained.

### 2.2. Field soil sampling protocols

Wet rainy season, and dry cool winter season were seasons examined. Topsoil samples (0-15 cm) were collected from 23 land cover types (Fig. 1B) during dry season (January 2022), and wet season (August 2022). January, typically following the December month which has low rainfall, is considered part of the dry season, while August, characterized by a peak in rainfall before it begins to decline, marks the wet season (A1 in S1 file). Therefore, the beginning of January and the end of August were chosen as the dry and wet seasons, respectively. Tables 2 and 3 presents the existing environmental conditions during soil sampling for each season. At each sampling season, three random distant locations were selected for each land cover type (i.e., 3 replications) and soil samples were collected from each location (i.e., 3 samples from each land cover type) following the similar sampling protocol of [39]. This means that a total of 69 soil samples from the 23 land cover types were collected for each season. The study’s small sample size of three replications may not adequately represent the entire sampling area; however, this limitation was due to constrained funding. All soil samples from both seasons were brought to the laboratory where they were air-dried and sieved at 2 mm-sieve for removing all identifiable plant residues prior to lab analysis.

**Table 2:** Soil and climatic conditions of the 23 land cover types during sampling season (dry season in January 2022, and wet season in August 2022).

S. No	Land cover types	Longitude	Latitude	Dry season				Wet season			
				T	SSW	SPM	P	T	SSW	P	SPM
1	Flatwood	−80.88361	27.65417	17.63	0.41	0.41	1.4	28.73	0.42	3.2	0.4
2	Mesic flatwood	−80.88667	27.65194	17.63	0.41	0.41	1.4	28.73	0.42	3.2	0.4
3	Dry hammock	−80.90111	27.64917	17.63	0.41	0.41	1.4	28.73	0.42	3.2	0.4
4	Xeric hammock	−80.96278	27.65611	16.71	0.32	0.32	1.18	28.51	0.44	4.82	0.37
5	Palm hammock (Xeric)	−80.95417	27.65722	16.71	0.32	0.32	1.18	28.51	0.44	4.82	0.37
6	Cypress	−81.00361	27.67611	16.71	0.32	0.32	1.18	28.51	0.44	4.82	0.37
7	Pinewood-1	−80.97750	27.65806	16.71	0.32	0.32	1.18	28.51	0.44	4.82	0.37
8	Hammock	−80.90389	27.64389	17.63	0.41	0.41	1.4	28.73	0.42	3.2	0.4
9	Pinewood-2	−80.89722	27.66194	17.63	0.41	0.41	1.4	28.73	0.42	3.2	0.4
10	Canal (wet)	−80.90167	27.64972	17.63	0.41	0.41	1.4	28.73	0.42	3.2	0.4
11	Wetland-1	−80.95417	27.65639	16.71	0.32	0.32	1.18	28.51	0.44	4.82	0.37
12	Wetland-2	−80.87778	27.66056	17.63	0.41	0.41	1.4	28.73	0.42	3.2	0.4
13	Wetland-3	−80.98472	27.69389	16.71	0.32	0.32	1.18	28.51	0.44	4.82	0.37
14	Wetland-4	−80.94583	27.65000	16.71	0.32	0.32	1.18	28.51	0.44	4.82	0.37
15	Sawtooth palmetto	−80.88667	27.65194	17.63	0.41	0.41	1.4	28.73	0.42	3.2	0.4
16	Sabal palmetto	−80.98472	27.69444	16.71	0.32	0.32	1.18	28.51	0.44	4.82	0.37
17	Dry Prairie-1	−80.90833	27.64944	17.63	0.41	0.41	1.4	28.73	0.42	3.2	0.4
18	Dry Prairie-2	−80.92833	27.64694	17.63	0.41	0.41	1.4	28.73	0.42	3.2	0.4
19	Dry Prairie-3	−80.89306	27.64278	17.63	0.41	0.41	1.4	28.73	0.42	3.2	0.4
20	Native pasture	−80.94361	27.65389	16.71	0.32	0.32	1.18	28.51	0.44	4.82	0.37
21	Improved pasture	−80.97306	27.71972	16.71	0.32	0.32	1.18	28.51	0.44	4.82	0.37
22	Abandoned citrus grove	−80.92500	27.67361	17.63	0.41	0.41	1.4	28.73	0.42	3.2	0.4
23	Sandy spoil	−81.02222	27.67722	16.71	0.32	0.32	1.18	28.51	0.44	4.82	0.37

T: Soil temperature (^o^ C) at 2 m; SSW: Surface soil wetness (dimensionless) with value of 1 indicates saturated soil conditions, while 0 indicates completely free water soils; SPM: Soil profile moisture from the surface down to the bedrock in m^3^ m^−3^; P: Monthly precipitation (mm/day). This is 2022 Dataset that was extracted from NASA POWER using geographical coordinates of each land cover (https://power.larc.nasa.gov/data-access-viewer/).

**Table 3:** Soil and climatic conditions of the 23 land cover types during sampling season (dry season in January 2022, and wet season in August 2022).

S. No.	Land cover types	Longitude	Latitude	Dry season				Wet season			
				Elevation (m)	GHI	ALB	S	Elevation (m)	GHI	ALB	S
1	Flatwood	−80.88361	27.65417	23.75	12.93	0.1	1	23.75	21.89	0.08	1
2	Mesic flatwood	−80.88667	27.65194	22.84	12.93	0.1	0.91	22.84	21.89	0.08	0.91
3	Dry hammock	−80.90111	27.64917	22.42	12.93	0.1	0.5	22.42	21.89	0.08	0.5
4	Xeric hammock	−80.96278	27.65611	28.46	12.93	0.1	1	28.46	21.89	0.08	1
5	Palm hammock (Xeric)	−80.95417	27.65722	21.24	12.93	0.1	1	21.24	21.89	0.08	1
6	Cypress	−81.00361	27.67611	36.02	13.16	0.15	0.78	36.02	21.31	0.12	0.78
7	Pinewood-1	−80.97750	27.65806	22.27	12.93	0.1	1	22.27	21.89	0.08	1
8	Hammock	−80.90389	27.64389	20.50	12.93	0.1	0.5	20.50	21.89	0.08	0.5
9	Pinewood-2	−80.89722	27.66194	23.06	12.93	0.1	0.83	23.06	21.89	0.08	0.83
10	Canal (wet)	−80.90167	27.64972	21.92	12.93	0.1	0.5	21.92	21.89	0.08	0.5
11	Wetland-1	−80.95417	27.65639	20.65	12.93	0.1	0.75	20.65	21.89	0.08	0.75
12	Wetland-2	−80.87778	27.66056	20.91	12.93	0.1	0.5	20.91	21.89	0.08	0.5
13	Wetland-3	−80.98472	27.69389	20.81	12.93	0.1	0.5	20.81	21.89	0.08	0.5
14	Wetland-4	−80.94583	27.65000	20.60	12.93	0.1	0.5	20.60	21.89	0.08	0.5
15	Sawtooth palmetto	−80.88667	27.65194	22.84	12.93	0.1	1	22.84	21.89	0.08	1
16	Sabal palmetto	−80.98472	27.69444	21.83	12.93	0.1	0.96	21.83	21.89	0.08	0.96
17	Dry Prairie-1	−80.90833	27.64944	21.62	12.93	0.1	1	21.62	21.89	0.08	1
18	Dry Prairie-2	−80.92833	27.64694	20.90	12.93	0.1	1	20.90	21.89	0.08	1
19	Dry Prairie-3	−80.89306	27.64278	22.59	12.93	0.1	1	22.59	21.89	0.08	1
20	Native pasture	−80.94361	27.65389	20.25	12.93	0.1	1	20.25	21.89	0.08	1
21	Improved pasture	−80.97306	27.71972	20.90	12.93	0.1	1	20.90	21.89	0.08	1
22	Abandoned citrus grove	−80.92500	27.67361	23.64	12.93	0.1	1	23.64	21.89	0.08	1
23	Sandy spoil	−81.02222	27.67722	19.36	13.16	0.15	1	19.36	21.31	0.12	1

GHI: Global Horizontal Irradiance (i.e., total solar radiation received on a horizontal surface in MJ/m2/day), ALB: Surface albedo (i.e., the fraction of solar radiation reflected by soil surface in dimensionless), S: slope gradient. This is 2022 Dataset that was extracted from NASA POWER using geographical coordinates of each land cover (https://power.larc.nasa.gov/data-access-viewer/). Elevations were extracted using Google Maps Elevation API (https://maps.googleapis.com/maps/api/elevation/json?locations=latitude, longitude &key=AIzaSyAkLj1kL2Y54LGuQXgvEx_JwiViYdpaeZY). Slope gradient was extracted from web soil survey (https://websoilsurvey.nrcs.usda.gov/app/) using geographical coordinates of each land cover.

### 2.3. Laboratory analyses

All soil samples were subjected to physicochemical analyses following standard procedures. Ten soil properties, including pH, bulk density (BD), maximum water holding capacity (MWHC), cation exchange capacity (CEC), organic matter (OM), active carbon (AC), soil protein (SP), total phosphorus (TP), total potassium (TK), and Total Kjeldahl Nitrogen (TKN) were analyzed. These soil indicators were considered as the total dataset (TDS) for soil health assessment. Soil pH (soil/water suspension of 1.5:15) was determined using an Accumet AB250 pH meter (Fisher Scientific, USA) [40]. To measure BD, a 25 ml graduated cylinder was levelled with oven dry soils, and then BD was quantified by dividing soil mass with 25 cm^3^ (volume of a cylinder) [41]. MWHC was measured by the modified protocol delineated by [42]. OM was determined based on the loss on ignition method at 600 ˚C. CEC was determined by employing the ammonium acetate method [43]. AC was measured using the K permanganate (KMnO_4_) method [44]. SP was measured using the sodium citrate extraction method [44] under autoclaving with high temperature and pressure. TP and TK were measured by ashing samples for at least 5 hrs (not to exceed 16 hrs) at 550 ˚C in a muffle furnace followed by extraction with 6 M HCl and analyzed using ICP-OES. TKN was analyzed using the Kjeldahl method [45]. The A2 in S1 file offers comprehensive details on how soil samples were analyzed. This contains step-by-step processes, detailed protocols, and illustrative images that explain each stage of the analysis. These resources intend to enhance understanding and facilitate replication of the study’s methods.

### 2.4. Comprehensive evaluation of soil health index, and its sensitivity to environmental factors

The soil health index (SHI) was evaluated through a three-step process, followed by a fourth step where changes in soil health status among seasons were assessed using SHI classification grades or classes (A3 in S1 file). The first step was to select a minimum dataset from the total dataset using loads and eigenvectors from Principal component analysis (PCA) [46]. The second step was to normalize the selected soil health indicators in the minimum dataset employing a standard scoring function (Eqs. 1 and 2), and the final step was to calculate the SHI (Eq. 4) based on the scores and weights of these indicators of soil health [6].

#### 2.4.1. Minimum dataset determination.

By performing Principal component analysis (PCA), the total dataset (TDS) with 10 soil indicators, such as pH, BD, MWHC, CEC, OM, AC, SP, TP, TK, and TKN, was reduced to a smaller set of independent (i.e., uncorrelated) indicators known as minimum dataset (MDS)—a set contains few variables that retain the most of information contained in the original TDS. MDS possesses sufficient information for SHI characterization [47]. PCA groups variables into different Principal components (PCs) (Table 4), and PCs with eigenvalues ≥ 1 that explained at least 5% of variation in the TDS were selected as desirable indicators [48]. We then selected only the soil variables with absolute load values within 10% of the highest variable load value in each PC [49]. Soil variables with load values ≥  0.30 in the PCs were retained as the most important contributors to their respective PCs for characterizing minimum dataset. In case the same soil variables that have a load value ≥  0.30 were found in multiple PCs, those variables were retained in the PC where they had a higher load value. When more than one variable was selected for one PC, Pearson correlation analysis was used for determining whether some variables should be removed to avoid data redundance [12–48]. If the retained variables were interrelated (*P* <  0.05), implying that those variables are redundant because of carrying the same information (i.e., perform similar soil functions), only the highest weighted variable was kept in the PC, and included in the MDS. If the retained variables were not correlated with each other (*P* >  0.05), implying that those variables carry different information (i.e., perform different soil functions), all those variables were kept in their respective PCs, and included in the MDS [6].

**Table 4:** Results of principal component analysis (PCA) of the selected indicators under dry and wet season.

Soil variables	Dry Season		Wet Season	Dry Season	Wet season
	PC1	PC2	PC1	Commonality	Commonality
PH	−**0.306**	−**0.374**	0.269	0.234	0.072
BD	**0.340**	0.055	**0.324**	0.118	0.105
OM	−**0.339**	0.060	−**0.331**	0.119	0.109
TK	−**0.337**	0.121	−**0.327**	0.128	0.107
TP	−**0.338**	−0.106	−**0.312**	0.125	0.097
AC	−**0.340**	−0.052	−**0.331**	0.118	0.109
TKN	0.009	**0.857**	−**0.327**	0.735	0.107
SP	−**0.321**	0.281	−**0.328**	0.182	0.108
MWHC	−**0.338**	0.101	−0.297	0.124	0.088
CEC	−**0.340**	0.026	**0.311**	0.116	0.097
Eigenvalues	8.64	1.36	9.13		
Variance (%)	86.40	13.60	9.13		
Cumulative variance (%)	86.40	100	91.26		

Bold eigenvector values ( ≥ 0.30) correspond to the highly weighted variables within individual PCs that were considered for MDS construction. Bold-underlined values correspond to key soil health indicators chosen for MDS—the screening criteria are detailed in the section 2.4.1. pH: soil pH; BD: bulk density; OM: organic matter; TK: total potassium; TP: total phosphorus; AC: active carbon; TKN: Total Kjeldahl Nitrogen; SP: soil protein; MWHC: maximum water holding capacity; CEC: cation exchange capacity.

#### 2.4.2. Normalizing selected indicators in the minimum dataset.

A linear scoring functions (Eqs. 1 and 2) were employed to normalize the selected MDS indicators (i.e., key soil health indicators) [50]. These scoring functions play a role in converting the value of each key indicator of soil health into dimensionless value that ranges from 0 to 1 [50]. The normalized value of each indicator is known as the score of that indicator, which signifies each indicator’s contribution to soil health. Soil health indicators were normalized using three scoring functions (**“more is better”**, **“less is better”**, and **“optimum”**). If the value of each indicator increases with soil health enhancement (E.g., increases in soil OM is associated with improvement in soil health), then a scoring function **“more is better”** was used (Eq. 1). Conversely, a scoring function of **“less is better”** was employed when increase in soil indicator is believed to be deleterious to soil health (Eq. 2) [50], for example, increases in soil BD is associated with increases in soil compaction, which in turn decrease soil health. Therefore, decreases in soil BD is associated with decreases in soil compaction, which in turn leads to soil health improvement, being the reason why **“less is better”** scoring function is applied to BD, and other parameters.

For **“more is better”**, value of each indicator (X) was divided by the highest observed value (Xmax) such that the highest value obtained a score equal to 1, while the rest values obtained a score less than 1.


$${\text{LSF=X/X}}_{\max}$$ (Eq. 1)

For **“less is better”**, the lowest observed value (Xmin) was divided by each observed value (X) such that the lowest observed value obtained a score equal to 1, while the rest value obtained the score less than 1.


$${\text{LSF=X}}_{\min}/\text{X}$$ (Eq. 2)

where LSF stands for linear score of each soil indicator of MDS lying between 0 and 1, X is the laboratory measured value of each indicator, and the higher and lower value of each measured soil health indicator is represented by X_max_ and X_min_, respectively. Soil health improvement is generally associated with (i) increasing in MWHC, CEC, OM, AC, SP, TP, TK, and TKN, and (ii) decreasing in BD— a reason why the **“more is better”**, and **“less is better”** scoring functions were used for these variables, respectively [51]. The optimum scoring function was used for only soil pH with threshold value ranging from 6.5-7.5 [51]; soil pH was scored as “**more is better**” up to the threshold value (i.e., 6.5), assigned a score of 1 when soil pH lies in the optimum range (i.e., 6.5–7.5), and then scored as “**less is better”** when soil pH’ values are greater than threshold value (i.e., >  7.5) [51–53].

#### 2.4.3. Assigning weights to each indicator.

Each soil attribute explains some amount of variance in the TDS [54]. PCA results were used to calculate each indicator’s weight based on its commonality value varying from 0 to 1 (Table 4), which indicates how each indicator contributes to the overall variance: the higher the commonality value, the higher contribution of indicator to overall variance [6]. Weights were calculated using the following formulae:


$$Wi=Ci/\overset{n}{\underset{i=0}{\sum }}Ci$$ (Eq. 3)

Where *Wi* stands for the weight of each indicator, *C*_*i*_ stands for the indicator’s commonality value derived from indicators’ eigenvector values, and *n* stands for the total number of indicators contained in the minimum dataset [47].

#### 2.4.4. Calculating SHI.

A SHI is used to evaluate changes in soil health [55]. In this study, we used SHI to assess changes in soil health between seasons in different land cover types. We computed the SHI following similar formulae as described by [47]:


$$SHI=\overset{n}{\underset{i=0}{\sum }}WiSi$$ (Eq. 4)

Where Si stands for normalized score of each indicator, *and Wi* stands for the weight of each indicator.

The fourth step is to assess soil health status among seasons based on SHI value. Pursuant to the SHI value, a higher SHI value corresponds to a higher level of soil health. According SHI classification criteria [6], SHI is grouped into 5 grades: very high (grade I, SHI ≥  0.85), high (grade II, 0.85 ≥  SHI ≥  0.7), medium (grade III, 0.7 ≥  SHI ≥  0.55), low (grade IV, 0.55 ≥  SHI ≥  0.4), and very low (grade V, SHI <  0.4), and we used these grades to evaluate how seasonal change affect soil health status under different land cover types. After characterizing SHI, linear regression analysis was performed to explore the sensitivity of SHI to environmental factors such as precipitation, soil temperature, surface soil wetness, soil profile moisture, elevation (El), slope gradient (S), global horizontal irradiance (GHI) and surface albedo (ALB).

### 2.5. Correlation among soil indicators, and their spatial distribution across seasons

To further comprehend how seasonal change shapes the relationships between soil indicators, Pearson correlation analysis was performed using R statistical software (version 4.2.3). PCA biplot was generated to understand how soil indicators’ spatial distribution across different land covers responds to seasonal change.

### 2.6. Statistical analysis

Principal component analysis was performed to remove redundancy from these evaluated soil indicators, and the obtained nonredundant soil indicators (i.e., MDS) were used for quantifying SHI. A paired sample t test was conducted to indicate the differences in soil indicators across different land cover types between seasons. Significant differences among means were assessed using Tukey’s Test at 5% significant level. Benjamini-Hochberg correction was applied to adjust the p-values to reflect the reality of multiple comparisons through reducing the risk of false significant results. Linear regression analysis was performed to understand the sensitivity of soil health to environmental factors. All analyses were conducted using R statistical software (version 4.2.3).

## 3. Results

### 3.1. Changes in soil indicators between seasons across different land cover types

**Fig 2:**
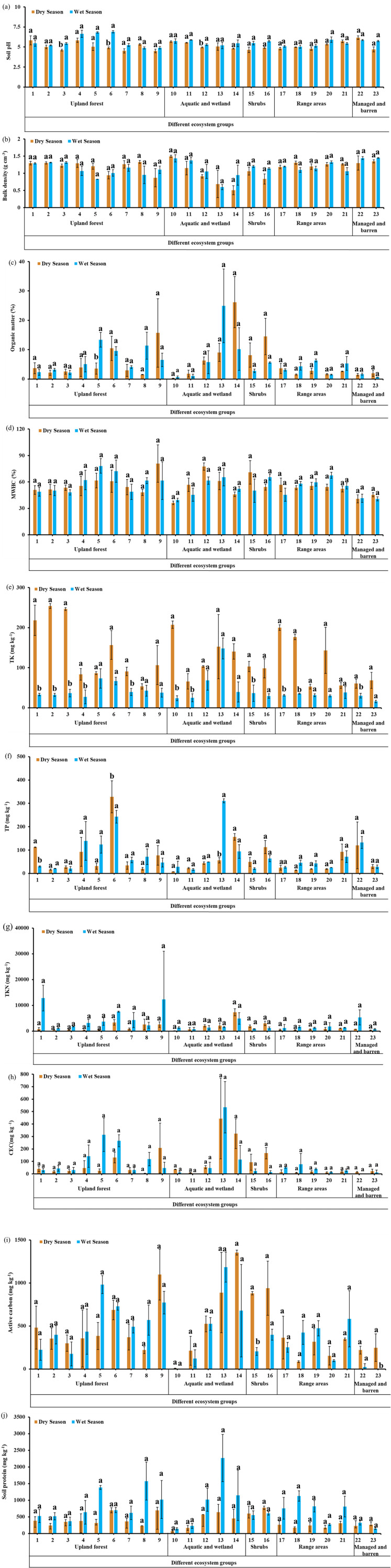
Impact of rewetting of dry soils on soil health indicators under 23 land cover types across different ecosystems groups. (a) soil pH, (b) bulk density, (c) organic matter, (d) maximum water holding capacity, (e) total potassium, (f) total phosphorous, (g) active carbon, (h) total Kjeldahl nitrogen, (i) cation exchange capacity, and (j) soil protein. Means with different letters in column are significantly different (*P* < 0.05), while the ones with same letters are nonsignificant (P > 0.05) pursuant to Tukey test.

The statistical comparison of physicochemical soil indicators of the dry and wet season showed that all evaluated soil indicators, including pH, BD, OM, MWHC, TK, TP, TKN, AC, SP, and CEC either increased (*P <  0.05*), declined (*P <  0.05*) or remained unaltered (*P >  0.05*) in the wet vs. dry season, depending on land cover type (Fig. 2). Soil pH was significantly (*P <  0.05)* higher in the wet than dry season under land cover type # 3, 6, and 12, while in the rest land cover types, soil pH remained unaltered (*P >  0.05*) between seasons. OM was significantly (*P <  0.05*) higher in the wet than dry season only in the land cover type #5, with no significant changes across seasons observed in the rest of land cover types (Fig. 2). TP was significantly (*P <  0.05*) lower in wet than dry season under the land cover # 1, and 6, while under land cover #13, TP was significantly (*P <  0.05*) higher in wet than dry season, with no significant difference in TP observed between seasons for the rest of land cover types. TK significantly (*P <  0.05*) was lower in wet than dry season under land cover # 1, 2, 3,4, 7, 10, 11, 15, 17, 18, and 22, with no significant difference observed in TK for the rest of land covers (Fig. 2). AC was significantly (*P <  0.05*) lower in wet than dry season under land cover # 15, and 23, with no observed significant changes in AC between seasons for the rest of land cover types (Fig. 2). Surprisingly, no significant differences (*P >  0.05*) in BD, MWHC, TKN, CEC, and SP were observed between seasons for all 23 evaluated land cover types (Fig. 2). Overall, changes in soil indicators between seasons, depended on land cover type.

### 3.2. Changes in correlation among soil indicators, and their spatial distribution across seasons

**Fig 3:**
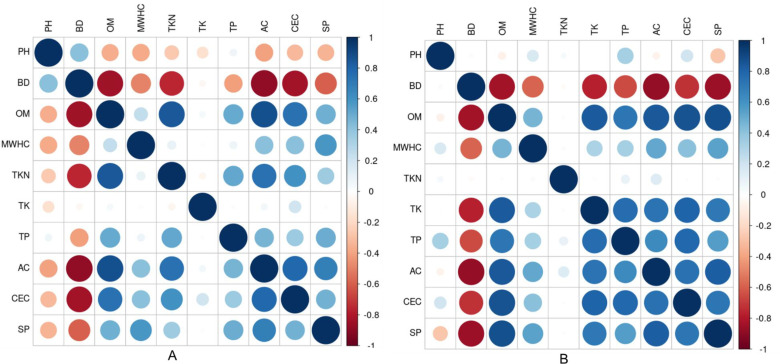
Changes in the relationship between soil indicators across seasons. A: Dry seasons; B: Wet season.

**Fig 4:**
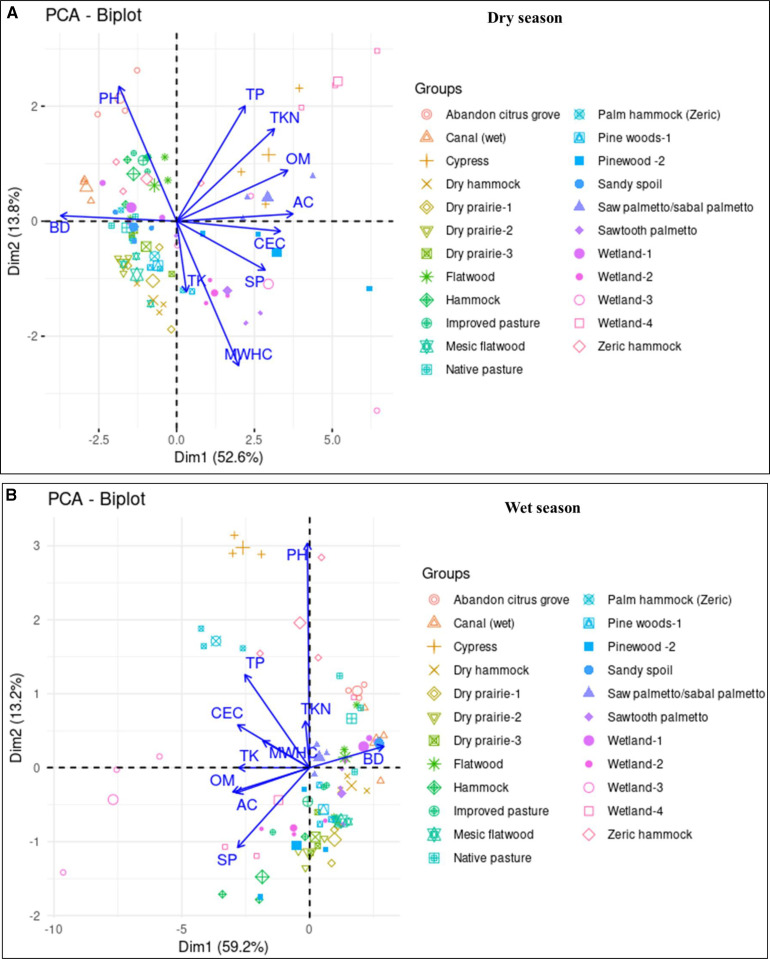
(A) Spatial distribution of soil indicators across different land cover types in the dry season using the first Two PCs. Groups stands for different 23 land covers involved in the study. Different symbols in different colors in the ordinations represent individual land cover. Big symbols in the ordinations indicate averages. (B) 4B. Spatial distribution of soil indicators across different land cover types in the wet season using the first two PCS. Groups stands for different 23 land covers involved in the study. Different symbols in different colors in the ordinations represent individual land cover. Big symbols in the ordinations indicate averages.

Pearson’s correlation analysis was performed between all evaluated soil indicators for both seasons. Results indicated that some soil indicators showed a relatively stable correlations, but others have shifted in intensity and even direction in the wet season vs. dry season (Fig. 3A and B). For instance, OM showed a weak positive correlation with TK in the dry season, which was strengthened in the wet season. Similarly, TP, and TK showed almost no correlation under dry season, while under wet season, they both displayed a strong positive correlation. CEC was negatively correlated with pH in the dry season, while under wet season, it was positively correlated with pH, indicating that the direction of correlation has shifted from negative to positive. Changes in soil indicators’ relationship between seasons (Fig. 3A and B), were also indicated by PCA biplot (Fig. 4A and B). There were changes in spatial distribution of soil indicators among seasons (Fig. 4A and B). PCA biplot showed that PC1, and PC2 respectively explained 52.6, and 13.6% of the variance, providing a cumulatively about 66.2% of total variance under dry season, while under wet season, PC1, and PC2 respectively explained 59.2, and 13.2% of the variance, giving cumulatively 72.4% of total variance (Fig. 4A and B). PCA biplot also revealed a shift in the relationship among land cover types between seasons (Fig. 4A and B). For instance, cypress and sabal palmetto land cover types were clustered together to the right side of PC1 based on TKN, OM, and AC in the dry season, while in the wet season, the same land covers were clearly separated from each other whereby cypress land cover shifted to the left side of PC1, and sabal palmetto land cover to the right side of PC1. The land covers were more spread out in the wet compared to the dry season, indicating more distinct differences in how land cover types respond to seasonal change (Fig. 4A and B).

### 3.3. Changes in SHI between seasons across different land cover types, and its sensitivity to environmental factors

For each of land cover type, a total of 10 soil health indicators, including physical and chemical indicators were considered for PCA and screened for calculating SHI. Based on the PCA results, there was two PCs for dry, and one PC for wet season; all PCs had eigenvalue >  1 and variance >  5% (Table 4).

**Fig 5:**
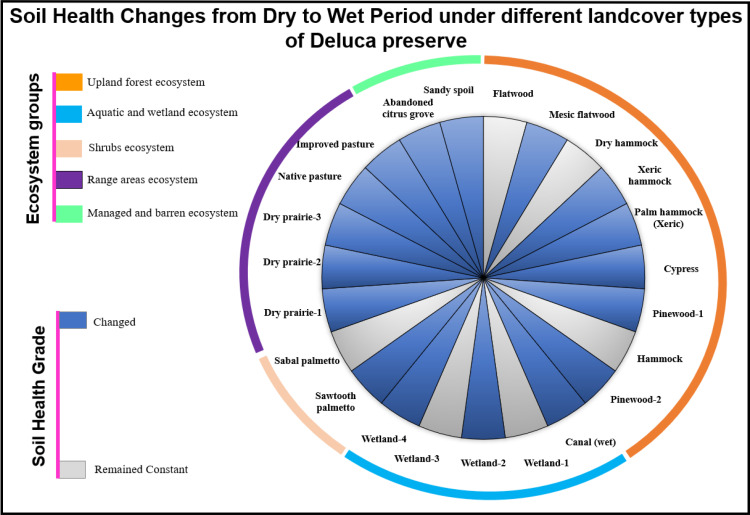
Changes in soil health grades between seasons under different land cover types across different ecosystem groups.

Based on SHI results, SHI value was either higher or lower in the wet season than in dry season, depending on land cover type (Table 5). By using SHI classification grades, soil health status either changed or remained stable between seasons, depending on land cover type (Table 5 , Fig. 5), with only poor soil health status (i.e., grade IV) being observed in the canal (wet) and dry prairie-1 land cover types (Table 5). Linear regression models were adopted to explore the stability of soil health to environmental factors, such as precipitation, soil temperature, soil profile moisture, surface soil wetness, elevation, solar radiation, slope gradient, and surface albedo. Out of eight evaluated environmental factors, only slope gradient significantly (*P <  0.05*) exhibited negative relationship with SHI for wet season (Table 6). In the dry season, non-significant negative relationships were observed between SHI and environmental factors, such as elevation, slope gradient, GHI, and ALB, while non-significant positive relationships were noted with surface soil wetness, precipitation, temperature, and soil moisture profile was observed (Table 6, *P >  0.05*). In the wet season, SHI showed non-significant negative relationships with precipitation, surface soil wetness, and elevation, whereas non-significant positive relationships were observed with soil temperature, soil moisture profile, and solar radiation (Table 6, *P >  0.05*). Overall, all models have a very low R² values (Table 6) for both seasons, indicating that the models explained minimal variability in SHI.

**Table 5:** SHI alongside their corresponding soil health classification grades in the different land cover types across various ecosystem groups between seasons.

Land cover types	Dry Season		Wet Season		Ecosystem groups
	SHI	Grade	SHI	Grade	
Flatwood	0.90	I	0.88	I	**Group A** **Upland Forests** (Forested Ecosystems/ Natural Tree Cover)
Mesic flatwood	0.78	II	0.90	I	
Dry hammock	0.81	II	0.83	II	
Xeric hammock	0.70	III	0.72	II	
Palm hammock (Xeric)	0.79	II	0.91	I	
Cypress	0.81	II	0.93	I	
Pinewood-1	0.81	II	0.88	I	
Hammock	0.84	II	0.75	II	
Pinewood-2	0.75	II	0.63	III	
Canal (wet)	0.89	I	0.41	IV	**Group B** **Wetland Ecosystems** (Wetland, Watercourse, and Waterbody Areas)
Wetland-1	0.82	II	0.76	II	
Wetland-2	0.91	I	0.79	II	
Wetland-3	0.73	II	0.84	II	
Wetland-4	0.81	II	0.66	III	
Sawtooth palmetto	0.87	I	0.80	II	**Group C** **Shrub Ecosystems**
Sabal palmetto	0.81	II	0.75	II	
Dry Prairie-1	0.73	II	0.54	IV	**Group D** **Range Areas** (Grassland and Prairie Ecosystems)
Dry Prairie-2	0.90	I	0.77	II	
Dry Prairie-3	0.81	II	0.86	I	
Native pasture	0.75	II	0.90	I	
Improved pasture	0.91	I	0.78	II	
Abandoned citrus grove	0.86	I	0.67	III	**Group E** **Barren Ecosystems**
Sandy spoil	0.79	II	0.69	III	

Soil health grades: very high (Grade I, SHI ≥  0.85), high (Grade II, 0.85 ≥  SHI ≥  0.7); medium (Grade III, 0.7 ≥  SHI ≥  0.55), low (Grade IV, 0.55 ≥  SHI ≥  0.4), and very low (Grade V, SHI <  0.4).

**Table 6:** Relationship between SHI, and environmental factors across seasons.

Seasons	Linear Models	R^2^	P value
Dry	SHI = 0.8792–0.1709SPM	0.06145	> 0.05
	SHI = 1.10387–0.01672T	0.01645	> 0.05
	SHI = 0.9070–0.0699P	0.01645	> 0.05
	SHI = 0.8792–0.1709SSW	0.06145	> 0.05
	SHI = 0.6555^ + ^0.0071El	0.1609	> 0.05
	SHI = –0.9396^ + ^0.1356GHI	0.02153	> 0.05
	SHI = 0.75143^ + ^0.6238ALB	0.00.2153	> 0.05
	SHI = 0.819612–0.003696S	0.0002	> 0.05
Wet	SHI = –0.5206^ + ^3.3351SPM	0.16	> 0.05
	SHI = –12.2526^ + ^0.4548T	0.16	> 0.05
	SHI = 1–0.062P	0.16	> 0.05
	SHI = 2.915–5.003SSW	0.16	> 0.05
	SHI = 0.9393–0.0077El	0.04306	> 0.05
	SHI = –0.68054^ + ^0.06622GHI	0.00750	> 0.05
	SHI = 0.98415–0.26144S	0.1964	< 0.05

SHI: Soil health index; SPM: Soil profile moisture; T: soil temperature: Precipitation; SSW: Surface soil wetness; El: Elevation (m); GHI: Global Horizontal Irradiance (i.e., total solar radiation received on a horizontal surface in MJ/m^2^/day); ALB: Surface albedo (i.e., the fraction of solar radiation reflected by soil surface in dimensionless); S: slope gradient.

## 4. Discussion

### 4.1. Changes in soil indicators between seasons across different land cover types

Usually, soil physical, chemical, and biological reactions that involve in the mineralization of soil elements [56–57] are influenced by environmental factors, such as soil temperature [58–59], moisture availability [60], rainfall [61], elevation [62], slope gradient [63], solar radiation [64], and surface albedo [65]. However, the response of soil indicators to these reactions depends on how soil type, and vegetation cover respond to these environmental factors’ variability. In the subtropical preserve investigated in this study, there is variability in soil temperature, moisture, precipitation, solar radiation, and surface albedo among seasons (Tables 2 and 3), and obviously, we observed either significant (P <  0.05) or insignificant (P >  0.05) differences in soil indicators between seasons, depending on land cover type (Fig. 2). In some land cover types, soil indicators were either significantly (P <  0.05) or insignificantly (P >  0.05) higher in the wet than dry season, while in the other land cover types, soil indicators were either significantly (P <  0.05) or insignificantly (P >  0.05) lower in the wet than dry season (Fig. 2). For each season, environmental factors, such as soil temperature, moisture, precipitation, elevation, solar radiation, and surface albedo were almost same for all 23 land cover types (Tables 2 and 3) since the subtropical preserve investigated in this study, is connected to one weather station. This means that changes in soil indicators among seasons in different land cover types obviously depended how site-specific soil type, and vegetation cover responded to seasonal change in soil temperature, moisture, precipitation, solar radiation, and surface albedo. For instance, OM was significantly (P <  0.05) higher in wet than dry season under land cover type #5, with no significant difference (P >  0.05) observed among seasons for the rest of land cover types (Fig. 2). The significant increase in OM is mainly due to the addition of new OM from oak tree leaves because Oak leaves and twigs are known to have high OM [66]. Our results are consistent with [67, 68] who also reported high OM under wet vs. dry season due to newly added plant debris. However, there was a decrease (P >  0.05) in OM in some land cover types under wet vs. dry season (Fig 2), reason being attributed to anaerobic OM decomposition, resulting in the conversion of initial OM to Methane gas (CH_4_) [67, 68, 69]. A downward or upward trends in BD under wet vs. dry season can be respectively ascribed to either increase or decrease in OM, observations that are consistent with previous studies [70–71]. There was a significant increase in soil pH during wet vs. dry season in the land cover # 3, 6, and 12, while no significant differences observed for rest of land covers (Fig. 2). This significant upward trends in soil pH can be ascribed to high levels of reduced iron under wet season vs. dry season that are responsible for rising soil pH [72]. Our results are in sync with previous studies conducted by [68–73], who reported that an increase in soil pH under wet vs. dry soil conditions. Though soil pH was insignificantly (P >  0.05) decreased under some land covers, these downward trends can be ascribed to accumulation of CO_2,_ and generation of high organic acids [67–72]. TK was significantly (P <  0.05) lower in wet than dry season for some land cover types, with no significant difference observed for other land covers (Fig. 2), implying that water movement in the topsoil as a result of high rainfall (Tables 2 and 3) leached out nutrients [74] because sandy soils in particular (Table 1) are prone to leaching. Similar trends were observed for TP where in some land cover types leaching effects reduced the concentration of TP during wet seasons (Fig. 2), an observation that agrees with previous report [75]. However, there was an increase in TP which was pronounced in the land cover #13 during wet season vs. dry season, which can be attributed reduction of Fe (III) to Fe (II) by iron-reducing bacteria, followed by releasing adsorbed and chemically bonded P in the soils [72–76]. Our results corroborate the findings of previous study conducted by [75], who reported that rewetting dry soils released P bound to soil colloids into the soil solution. AC is a fraction of soil OM that can be easily oxidized to CO_2_ by soil microbial respiration [40]. In some land cover types, AC decreased under wet vs. dry season with a pronounced decrease being observed in the land cover # 15, and 23 (Fig. 2). This downward trends in AC are mainly due to the decrease in soil C pool as Methane gas produced from anaerobic OM decomposition escape from the soil [72]. There were also nonsignificant increases in AC under wet season versus dry season (Fig. 2), reason being due to anaerobic soil conditions that reduced the rate at which soil microbes consume AC for getting energy [77], coupled with release of labile OM locked up inside of soil aggregates [30]. Our observations are in sync with the previous reports [78–79].

Though TKN, CEC, SP, and MWHC did not show any significant difference in all land cover types between seasons, there was upward and downward trends among seasons depending on land cover type (Fig. 2). TKN was higher in wet than in dry season (Fig. 2), probably due to nitrogen fixers such as algae and bacteria that perform well under anaerobic versus aerobic soil conditions [72–80], coupled with the addition of bioavailable organic N from newly added organic materials [81]. Our findings are consistent with previous studies conducted by [71], who reported a 32% increase in TKN under wet vs. dry season. On the other hand, reduction in TKN under wet vs. dry season was also observed in some land cover types (Fig. 2), probably due to increases in denitrification process [72–79] and NO_3_^-^ reduction [82–83], coupled with water movement in the topsoil that leaches out NO_3_, and NH_4_^ +^ [84]. CEC either increased or decreased in wet vs. dry season, depending on land cover type (Fig. 2); the observed upward trends in CEC can be ascribed to high OM (Fig. 2), while the downward trends in CEC may be ascribed to water movement in the topsoil that leached out basic cations [74]. SP is a fraction of organically bound N in soil OM that is susceptible to being mineralized by soil microorganisms [40] and is important because it is required by these soil organisms for their reproduction. Increases in SP under wet vs. dry season was observed in some land cover types (Fig. 2), reason being due to high OM content (Fig. 2). Our results are comparable with previous study conducted by [40], who reported a high SP in flooded vs. dry soil conditions. However, we also observed a decrease in SP under wet vs. dry season in some land cover types (Fig. 2), probably due to denitrification process under anaerobic conditions [77–82], which led to a reduction in soil organic N pool. There was the increase in MWHC in some land cover types under wet vs. dry season, due to increases in OM (Fig. 2), observations that are consistent with previous studies [40,85,86]. However, we also observed MWHC reductions in some land cover types when OM increased (Fig 2), reason for this is unclear, but one study reported that OM has little or no influence on MHWC [84]. Overall, our results propose that response of soil indicators to seasonal change depends obviously on-site specific soil types, and land cover types. This is also supported by our PCA biplot where the land covers were more spread out in the wet compared to the dry season (Fig. 4A and B ), indicating more distinct differences in how land cover types respond to seasonal change. The results of this study provide two key implications. First, the lack of significant changes in most of soil indicators across seasons suggests that seasonal variation has minimal influence on these indicators. This finding underscores the need for further research focusing on different soil metrics, such as microbial indicators, which are known to be more sensitive to short-term soil management practices. Second, the non-significant findings may be attributed to the study’s small sample size (i.e., three replications), a limitation stemming from constrained funding. This highlights the importance of future research incorporating larger sample sizes to validate our findings.

### 4.2. Changes in correlation among soil indicators, and their spatial distribution between seasons

Previous research has indicated that soil physical, chemical, and biological reactions that involve in the oxidation of soil elements [56–57] are influenced by soil temperature [58–59], moisture availability [60], and rainfall [61]. In the study area, there was variability in soil temperature, moisture, and rainfall among seasons (Tables 2 and 3), thereby affecting the way soil indicators relate to each other among seasons, as well as their spatial distributions. Fig. 3A and B showed that some soil indicators displayed a relatively stable correlations, but others have shifted in intensity and even direction, suggesting that the relationships between these soil indicators have changed across seasons. PCA biplot (Fig. 4A and B) also indicated that seasonal change shifted the relationship between soil indicators, and their spatial distribution across different land covers. These shifts in intensity, direction, and spatial distribution across seasons are potentially due to changes in environmental conditions between seasons (Tables 2 and 3). Another potential reason for our results may be due to biogeochemical equilibrium, nutrient input, and output, which could have changed during the annual, cyclical hydrologic regime, thereby influencing OM, and soil nutrient content [68], and in turn affect how soil indicators relate to each other as well as their spatial distribution across different land covers.

### 4.3. Changes in SHI between seasons, and its sensitivity to environmental factors

A high SHI value corresponds to high soil health, while a low SHI value represents low soil health or soil health deterioration [42]. Our results showed that SHI was either higher or lower in wet than dry season, depending on land cover type (Table 5), an observation that is consistent with previous studies [55] where soil health varied, depending on vegetation type. For example, land cover type #2, 3, 4, 5, 6 and 7 exhibited a higher value of SHI under wet vs. dry season. This is mainly since these land covers are grouped into upland forest ecosystem (Fig 1B) where new OM from dead plant materials including leaves, bark, needles, twigs, etc... fall to the ground, and subsequently improving soil health. The role of OM builds up in improving soil health has been previously reported. For instance, OM buildup improves soil health through soil structure improvement [87], enhancing nutrient storage, and supply [88], increasing water retention capacity [89], and increasing microbial activities [90]. On the hand, SHI value was lower in wet than dry season in some land covers (E.g., land cover # 22, and 23). These findings were expected because these land covers are grouped into barren ecosystem characterized by almost exposed soils due to less vegetation. Under high rainfall events (i.e., wet season), nutrients get leached out, in addition to lack of new OM addition, thereby decreasing soil health. Since soil environmental factors were almost same in all land cover types for each season, this inconsistent trends on SHI between seasons across different land cover types is ascribed to how site-specific soil type, and vegetation cover respond to variability in environmental factor such as precipitation, soil temperature, moisture, soil radiation, elevation, slope gradient, and surface albedo. The influence of these environmental factors on soil health has been reported in many studies; for instance, soil moisture influences soil health by controlling biological and biogeochemical processes [91] and influencing microbial dynamics and many soil chemical/physical properties [91]. However, out of 8 environmental factors evaluated in this study, we only observed a significant relationship between SHI, and slope gradient during wet season, with non-significant relationship observed for other environmental factors (Table 6 ), indicating that slope gradient is the main factor influencing soil health within non-agronomic landscape due to its influence on water retention, and flow The impact of slope gradient on soil indicators, and overall soil health has been previous studied. For instance, [92] reported that high slope gradient decreased SHI; [93] revealed that steeper slopes have highest soil compaction, and lowest total porosity, which in turn reduces water, and nutrient retention and subsequently decrease soil health; [94] observed changes in soil quality due to variations in topography and slope gradient; [95] found a decline in soil nutrient level under upper slope region relative to lower slope area due to soil loss by erosion; [96] observed OM losses in upslope area gradient with OM accretion under gentle slope areas and depression areas; [93] reported variations in soil health indicators due to changes in slope gradient, and land use and management; and [93] revealed that soil indicators including CEC, exchangeable magnesium, TKN, M3-P, and M3-K exhibited negative relationship with slope gradient. These previous findings align with our results, where slope gradient emerged as the primary environmental factor influencing soil health in the study area. The regression model demonstrated that an increase in slope gradient leads to a decrease in soil health. Our findings indicate that site-specific land cover type combined with soil features particularly slope gradient are attributable to controlling SHI over other environmental factors, observations that are in line with [79,93,97,98] who reported that vegetation, and slope gradient play role in controlling soil health through increasing soil OM, and nutrients. A decrease in SHI could be ascribed to either the water movement within the topsoil layer that leached out soil health indicators [99–101] or nutrient removal from the system by vegetation. Overall, the result of this study confirms that increasing in slope gradient can potentially decrease soil health within non-agronomic landscapes due to its influence on water storage, and flow, thereby affecting soil health indictors.

Based on the SHI classification grades [6], we found that soil health status either changed or did not change under wet vs. dry season, depending on land cover type (Fig. 4), with only soil health being worsened in the canal (wet) land cover type (i.e., from grade I: very high soil health — under dry season to grade IV: poor soil health — under wet season) and dry prairie-1 land cover types (i.e., from grade II: high soil health — under dry season to grade IV: poor soil health — under wet season) (Table 5). This change in soil health grade under wet vs. dry season can be ascribed to either water movement within the topsoil layer that might have decreased the concentration of soil health indicators via leaching [72,83,101] or increased the concentration of soil health indicators through mineralization of organic material [79-97]. From the results of this study, we can conclude that seasonal change impact soil health, depending on site-specific land cover types, and slope gradient rather than other selected environmental factors (SPM, T, P, SSW, El, HDI, and ALB). This study covered a small sample size due to limited funding, suggesting future research incorporating larger sample size to validate the findings of this study.

### 4.4. Constraints, and looking forward

This research has obtained reliable findings, which were comprehensively discussed. In this study, we used a small sample size (i.e., three replications), due to difficulties in accessing every part of the 23 land cover types that were evaluated in this study, a limitation stemming from limited funding. However, there may be two potential suggestions if future condition permits. First suggestion is that large sample size may offer more information in soil health evaluation, a suggestion that agrees with previous reports [36]. Increasing sample size can enhance the universality of the findings. We could not consider soil microbial indicators because our proposal aim was to focus on physicochemical soil indicators. So, the second suggestion is that microbial soil indicators should be incorporated in the TDS of soil health evaluation as their inclusion may improve the selection ability of MDS and sensitivity of soil health to environmental factors.

## 5. Conclusions

This study investigated how soil indicators, their relationship and overall soil health changes between dry and wet seasons across different land cover types and explored sensitivity of soil health to environmental factors. Our results showed that soil indicators, including pH, BD, MWHC, CEC, OM, AC, SP, TP, TK, and TKN either increased (P <  0. 05), decreased (p <  0. 05) or remained constant (P >  0. 05) under wet vs. dry season, depending on land cover type. Correlation analysis indicated that some soil indicators displayed a relatively stable correlations, but others have shifted in intensity and even direction in the wet vs. dry season. Based on PCA biplot, the land cover types were more spread out in the wet vs. the dry season, indicating more distinct differences in environmental characteristics among land covers between seasons, which in turn shifted spatial distribution of soil indicators. SHI either increased or decreased under wet vs. dry season depending on land cover types. Using SHI classification grade to categorize soil health status, results showed that soil health status either changed or did not change under wet vs. dry season, depending on land cover type, highlighting the dominant influence of site-specific land cover types on controlling soil health. The linear regression analysis indicates that the 8 selected environmental factors (SPM, T, P, SSW, elevation, GHI, slope gradient, and surface albedo) do not significantly explain variations in soil SHI for either the dry or wet seasons, except for slope gradient, which significantly influenced SHI during the wet season. This highlights slope gradient as the primary factor affecting soil health within non-agronomic landscapes due to its impact on water storage and flow. The results of this study identified two main implications. First, the lack of significant changes in most soil indicators across seasons suggests that seasonal variation has minimal influence on these indicators, underscoring the need for further research focusing on different soil metrics, such as microbial indicators, which are known to be more sensitive to short-term soil management practices. Second, the non-significant findings among indicators across seasons may be attributed to the study’s small sample size (i.e., three replications), a limitation stemming from constrained funding. This highlights the importance of future research incorporating larger sample sizes to validate our findings.

## Supporting Information

S1 File Supporting Information
(DOCX)
