# Supplementary material for: Responses of soil health to seasonal change under different land cover types in a sub-tropical preserve ecosystem
Source: PLoS One. 2025 Mar 25;20(3):e0318092. doi: 10.1371/journal.pone.0318092 (PMC11936207; doi:10.1371/journal.pone.0318092)
Supplement: S1 File — (DOCX) [file pone.0318092.s001.docx]

**Supplementary Materials**

| PARAMETER | JAN | FEB | MAR | APR | MAY | JUN | JUL | AUG | SEP | OCT | NOV | DEC |
| --- | --- | --- | --- | --- | --- | --- | --- | --- | --- | --- | --- | --- |
| T2M_MAX (^o^ C) | 30.19 | 32.045 | 35.245 | 36.725 | 38.605 | 39.245 | 38.89 | 36.125 | 35.05 | 33.425 | 31.505 | 30.76 |
| T2M_MIN (^o^ C) | -5.815 | -2.915 | 0.595 | 3.53 | 10.41 | 14.25 | 21.16 | 20.315 | 16.055 | 5.1 | 3.9 | -6.085 |
| Rainfall (mm) | 2173.105 | 2290.835 | 3118.815 | 2551.195 | 3833.05 | 8060.2 | 7176.195 | 7832.15 | 6993.79 | 3885.72 | 2330.76 | 1927.525 |

**A1.** The Study area’s monthly averaged Soil Temperature and Rainfall in the past 41 years from1981-2022.

T2M_MAX; Maximum soil temperature at 2 m; T2M_MIN: Minimum soil temperature at 2 m. Source: This Dataset was extracted from NASA POWER ( <https://power.larc.nasa.gov/data-access-viewer/> ), and display averaged data from 1981-2022.

**A2.** Laboratory protocol used for analyzing soil indicators

*pH*

Soil pH is measured using a 1.5:15 soil-to-deionized water ratio with an Accumet AB250 pH meter. In this method, 20 ml of deionized water is added to 10 g of mineral soil in a cup and mixed thoroughly with a stirring rod. After mixing, the samples are left to stand for 15 minutes to allow the soil to settle. The Accumet AB250 pH meter is then measured by placing the pH meter electrodes in the clear supernatant above the soil.

*Bulk Density (BD)*

BD is measured following the modified protocol developed by Jacobs et al. (1964). Oven-dry, disturbed soil sample is sieved through a 2 mm sieve and carefully added to a 25 ml graduated cylinder. The cylinder is filled up to one-fourth of its volume with the sieved soil, and natural packing is simulated by gently tapping the cylinder five times. The cylinder is then filled with more soil up to three-fourths of its volume, and the same tapping procedure is repeated to ensure consistent packing. Finally, the cylinder is completely filled with soil, and the BD is calculated using the following formula:

BD=Mass of soil (g)/Volume of soil (cm^3^)

*Cation exchange capacity (CEC)*

CEC is measured using the ammonium acetate method, as described by Chapman (1965) and Sumner and Miller (1996). In this method, soil (2g) is leached with an excess of neutral 1N ammonium acetate solution (20 ml) to displace exchangeable cations and saturate the exchange sites with ammonium ions. Excess ammonium is then removed by washing the soil with alcohol (15 ml) to eliminate any free ammonium. Subsequently, a 1N NaCl solution (20 ml) is used to release ammonium ions from the soil colloids into the soil solution. The concentration of retained ammonium ions in the supernatant is measured using the AQ 400 Instrument and is used to calculate the cation exchange capacity (CEC) in cmolc/kg. This is a brief method, and more details can be found in the cited document.

*Maximum water holding capacity (MWHC)*

MWHC was determined by the modified method described in Jenkinson and Powlson (1976) based on saturation procedure. In this method, 50 g oven dry mineral soil (25g of muck soil) are placed into a funnel with a Whatman No.5 filter paper (185 mm diameter) attached a rubber hose with clip closed, 50 mL of water (100 mL may be required for soils with high MWHCs) is added and then the soil is allowed to stand for 30 minutes. After this time, the clip is opened and allowed to drain for 15 minutes (sandy soil), or until it stops draining (for muck soil), and then the amount of water drained is measured. The difference between the volume of water added and the volume of water drained, minus the water held in the filter paper (1.98 ± 0.18 Sd ml), plus the water previously in the soil, is taken as water holding capacity (MWHC).

MWHC % = ((50ml — water drained — 1.98 + water in soil previously)/50g) x 100

Note: Water held Whatman # 5 filter paper, Ave (STD) = 1.98 ml (0.18) (n=2)

For dry soil, water in soil previously = 0

*Soil Organic Matter (SOM)*

SOM content is determined using the loss on ignition method at 600˚C. In this method, 5 g of oven-dry soil is heated at 600˚C in a muffle furnace for 24 hours. After ignition, the percentage of organic matter (% SOM) is calculated using the following formula:

$\%SOM=\frac{Oven Weight-Furnace Weight}{Sample dry weight}$ x 100,

Where:

- Oven Weight is the combined weight of the beaker and the oven-dry soil before ignition.
- Furnace Weight is the combined weight of the beaker and the soil after ignition in the muffle furnace at 600˚C.
- Sample Dry Weight is the weight of the oven-dry soil (5 g) initially placed in the furnace.

*Active Carbon (AC)*

AC is determined using the potassium permanganate (KMnO₄) oxidizable carbon method, as described by Schindelbeck et al. (2016). For muck soils, 1.0 g of soil is reacted with 20 ml of 0.2 M KMnO₄ solution for exactly 2 minutes with a dilution factor of 40. For mineral soils, the protocol uses 2.5 g of soil with 20 ml of 0.02 M KMnO₄ solution, analyzed under the same conditions but with a dilution factor of 20. After the reaction, the mixture is filtered, and the supernatant is analyzed at 550 nm using a Thermo Scientific Genesys 30 spectrophotometer.

*Soil Protein*

Soil protein is determined using a sodium citrate extraction method (Schindelbeck et al., 2016) under autoclaving conditions with high temperature and pressure. In this method, 1.5 g of soil is placed in a 20 mL glass tube, and 12 mL of 0.02 M sodium citrate (adjusted to pH 7 with HCl) is added. The mixture is shaken for 5 minutes on a horizontal shaker. The samples are then autoclaved at 121°C for 30 minutes and shaken again for 3 minutes on a horizontal shaker. After shaking, the mixture is left to settle for 5 minutes. Approximately 1.75 mL of the extract is withdrawn and transferred to a 2 mL microcentrifuge tube, where it is centrifuged at 10,000 g for 3 minutes. Next, 90 μL of the purified extract is added to 1.8 mL of BCA reagent in a new 2 mL microcentrifuge tube. The samples, along with standards, are incubated at 60°C for 30 minutes in a hot air oven. After incubation, the samples are brought to room temperature and analyzed at 562 nm using a spectrophotometer within 10 minutes. The extracted protein is quantified using the Thermo Pierce Colorimetric Bicinchoninic Acid Assay (BCA), calibrated against protein standards of known concentrations.

*Total Kjeldahl Nitrogen (TKN)*

TKN is determined using the digestion method followed by colorimetric determination, as described in EPA Method 351.2. Approximately 0.2 g of the sample is combined with 2 g of Kjeldahl digestion mixture (potassium sulfate and copper sulfate) and 5 mL of concentrated sulfuric acid in a digestion tube. The sample is digested at 250°C for 1 hour, then at 365°C for an additional 2.5–3 hours, converting organic nitrogen into ammonium. After cooling, the digest is diluted to 35 mL, filtered, and prepared for analysis. The nitrogen content is determined using either the Shimadzu TOC-L combustion method or a colorimetric method. Results are calibrated against standards and expressed as mg/kg total nitrogen in the sample.

*Total Phosphorus (TP) and Total Potassium (TK)*

TP and TK are determined using a method adapted from the University of Florida, Institute of Food and Agricultural Sciences (UF/IFAS, 2014). Extension Soil Testing Laboratory Analytical Procedures and Training Manual, with modifications based on Soltanpour et al. (1996). For soil and plant tissue samples, 0.4 g of the sample is ashed in a muffle furnace at 500–550˚C for 5 hours (not exceeding 16 hours for extended ashing). After cooling the samples to below 200°C, 2 mL of 6M HCl is added, and the mixture is left to react for 2 hours. Subsequently, 18 mL of deionized water is added to the sample, and the solution is shaken to ensure homogeneity. The resulting extract is analyzed using Inductively Coupled Plasma Optical Emission Spectroscopy (ICP-OES) to quantify the concentrations of P and K.

**References**

Chapman, H.D. 1965. Cation‐exchange capacity. Methods of soil analysis: Part 2 Chemical and microbiological properties, 9, 891-901.

Jacobs, H.S., Reed, R.M., Thien, S.J., Withee, L.V. 1964. Soils laboratory exercise source book. Amer. Soc. of Agron., Madison, WI.

Jenkinson, D. S., Powlson, D. S. 1976. Effects of biocidal treatments on metabolism in soil V: A method for measuring soil biomass. Soil Biology and Biochemistry, 8, 209–213.

Schindelbeck, R.R., Moebius-Clune, B. N., Moebius-Clune, D. J., Kurtz, K.S., van Es, H.M. 2016. Cornell University comprehensiveassessment of soil health laboratory standard operating procedures. Cornell University.

Soltanpour, P. N., Johnson, G. W., Workman, S. M., Jones Jr, J. B., Miller, R. O. 1996. Inductively coupled plasma emission spectrometry and inductively coupled plasma‐mass spectrometry. Methods of Soil Analysis: Part 3 Chemical Methods, 5, 91-139.

Sumner, M. E., Miller, M. P. 1996. Cation exchange capacity and exchange coefficient. In D. L. Sparks (Ed.). Methods of soil analysis. Part 3. Chemical methods (2nd ed., pp. 1201–1230). ASA and SSSA.

University of Florida, Institute of Food and Agricultural Sciences (UF/IFAS). 2014. Soil testing methods for Florida soils. Retrieved from <https://ufdcimages.uflib.ufl.edu/IR/00/00/34/69/00001/SS31200.pdf>


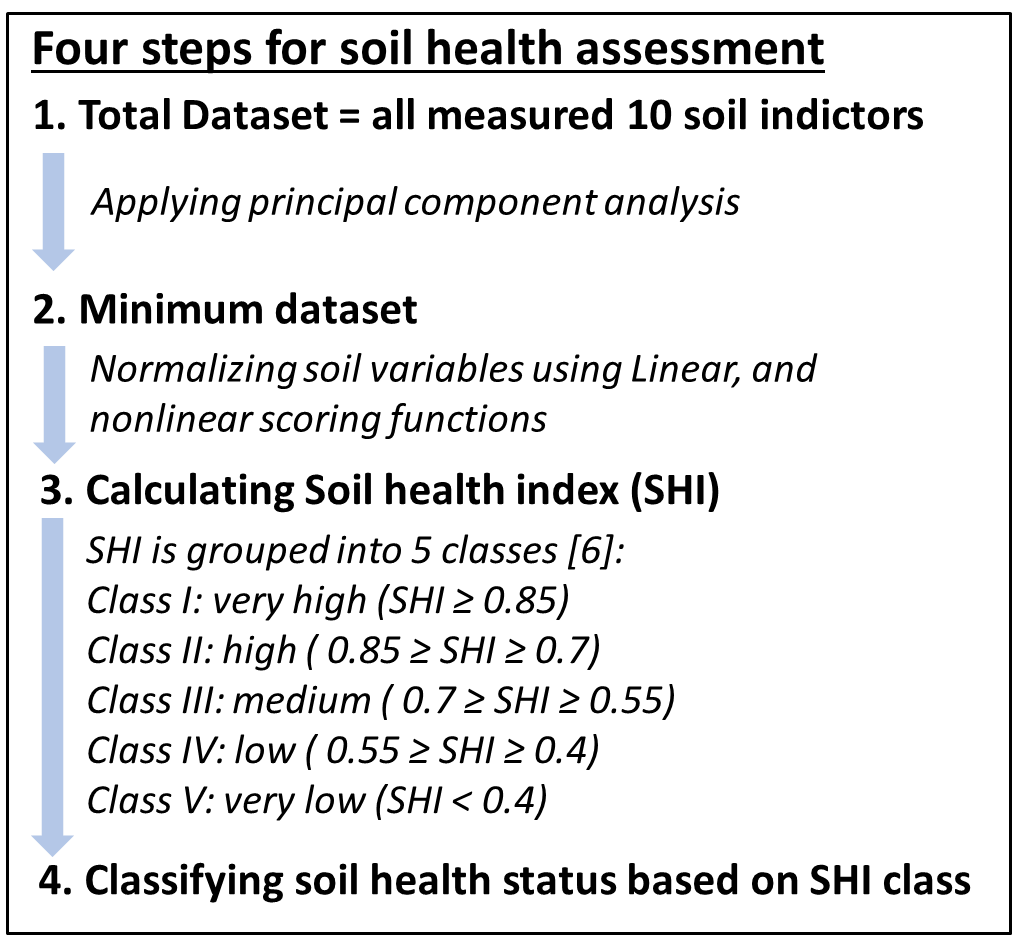


**A3.** Flowchart indicating various steps used for assessing soil health

**Note:** This flowchart of steps involved in soil health assessment. More details regarding each step are in the text from section 2.4.1-2.4.4.
